# Beam dynamic study of a Ka-band microwave undulator and its potential drive sources

**DOI:** 10.1038/s41598-022-11101-2

**Published:** 2022-04-29

**Authors:** Liang Zhang, Craig R. Donaldson, Jim Clarke, Jack Easton, Craig W. Robertson, Colin G. Whyte, Adrian W. Cross

**Affiliations:** 1 Department of Physics, University of Strathclyde, Glasgow, G4 0NG UK; 2 The Cockcroft Institute, Sci-Tech Daresbury, Keckwick Lane, Warrington, WA4 4AD UK; 3 ASTeC, STFC Daresbury Laboratory, Sci-Tech Daresbury, Keckwick Lane, Warrington, WA4 4AD UK

**Keywords:** Electronic and spintronic devices, Free-electron lasers

## Abstract

Microwave undulators (MUs) have great potential to be an alternative solution to permanent magnet undulators in a free electron laser (FEL) when shorter undulator periods are required. In this paper, the factors that affect the choice of the high-power drive sources were studied via a Ka-band cavity-type MU with a corrugated waveguide proposed for the CompactLight X-ray FEL. They include the technology of the high-power vacuum electronic devices, the quality factor of the MU cavity that was demonstrated by prototyping a short section of the MU structure, and the beam dynamic study of the electrons’ trajectories inside the MU. It showed that at high beam energy, a high-power oscillator is feasible to be used as the drive source. At low beam energy, the maximum transverse drift distance becomes larger therefore an amplifier has to be used to minimize the drift distance of the electrons by controlling the injection phase.

## Introduction

Since the free-electron laser (FEL) was first demonstrated by John Madey at Stanford in 1977^1^, its capability in producing high-power ultrashort-wavelength, and spatially coherent radiation opened various applications in biophysical and materials science, surface studies, chemical technology, medical applications, and solid-state physics. FELs with higher average power, higher repetition rate and shorter wavelengths at the X-ray frequency range have been in operation in recent years. They allow exploration of new studies in various fields of science that were not feasible before^2,3^.

The undulator is an essential component in an FEL. It interacts with the relativistic electrons to generate coherent radiation. Traditionally, an undulator is made of periodic permanent magnets^4,5^. The radiation wavelength produced by a permanent magnet undulator (PMU) is given by

1 $$\begin{aligned} \lambda = \lambda _u (1+k^2/2+ \gamma ^2 \theta ^2) / (2\gamma ^2), \end{aligned}$$

where $\lambda _u$ is the undulator period, which is the same as the period of the magnet, $\gamma$ is the relativistic factor, and $\theta$ is the observation angle. *k* is the undulator strength parameter defined by $k=0.0931\ B_0[\mathrm {T}]\lambda _u [\mathrm {mm}]$. Meanwhile, the gain of an FEL is determined by the dimensionless Pierce parameter $\rho$, which is

2 $$\begin{aligned} \rho ={\left( \frac{J^2}{\left( 8\pi \right) ^2}\frac{I_e}{I_A} \frac{k^2\lambda _u^2}{\gamma ^2\sigma ^2}\right) }^{1/3}, \end{aligned}$$

where *J* is the Bessel function factor, $I_e$ is the electron peak current, $I_A$=17 kA is the Alfven current and $\sigma$ is the RMS transverse size of the electron beam^4^.

Equation (1) shows short wavelength radiation can be achieved by either increasing the beam energy or reducing the undulator period. For example, to achieve $1\dot{A}$ (12.4 keV) radiation, the Swiss-XFEL has a beam energy of 5.8 GeV and an undulator period of 15 mm^6^. An acceleration section with a total length of 440 m is used to achieve the required beam energy. Higher beam energy requires a longer acceleration section, which is costly. The use of a short period undulator enables a low energy beam to produce radiation of a certain wavelength as compared to a longer period undulator which would require a higher energy beam. From Eq. (2), lower beam energy will also help to increase the Pierce parameter. An undulator with a short period and a high magnetic field is of great importance for a compact X-ray FEL facility. The state-of-the-art PMU used in the Swiss-FEL has a magnetic field strength of 1.29 T at the period of 15 mm. Due to the limit of the physical size of the permanent magnets, it is challenging to reduce the period of a PMU while keeping a large beam aperture and maintaining a high magnetic field. Superconducting magnets and cryogenic permanent magnets that can achieve higher magnetic fields attract great research interest and have made significant progress in recent years^7–9^.

The electromagnetic (EM) wave naturally has a periodic magnetic field. It can be used as an undulator if a suitable EM mode is chosen. One of the big advantages of the EM undulator is the relatively large aperture for the electron beam. The beam aperture can be of the same order as the period of the EM undulator, which is physically not possible in a PMU where the aperture must reduce as the magnet period reduces to achieve a reasonable magnetic field. The short undulator period of the EM undulator can be achieved through operating at a high frequency. A conceptual design showed that the laser-driven undulator could generate X-ray radiation with a MeV-level electron beam^10^. The challenge is the electron bunch requires ultralow-emittance and ultrashort bunch length. Another advantage of the EM undulator is the fast-electrical tuning capability. The magnetic field strength can be adjusted by the drive power, and the polarization can be controlled by the polarization of the input wave.

In the microwave frequency range, such as $\sim$ 10 GHz, its undulator period is close to the state-of-the-art PMU. A metallic cavity structure that generates a periodic transverse magnetic field through a standing wave was used in earlier studies^11^. A MU that employed a rigid rectangular waveguide at 2.856 GHz was developed to prove the operating principle in the 1980s. It achieved a magnetic field $B_u$ of 4.5 mT, resulting in a *k* factor of 0.24^12^. Its performance was limited by the high-power microwave source and microwave breakdown. Recent progress of the MU demonstrated a $B_u$ of 0.65 T and an undulator period of 13.9 mm operating at 11.424 GHz^13^. At a higher frequency of 91.392 GHz, a special MU structure was also designed to maintain a large beam aperture radius of 1.2 mm^14^.

In this paper, the power level needed to drive the MU and the potential high-power microwave sources to achieve multi-MW output power were investigated. Further detailed parameters were obtained from the design and the prototype of a MU operating at 36 GHz for CompactLight XFEL R&D^15–17^ are presented. The feasibility of using an oscillator as the drive source was also numerically studied from the beam dynamics inside the designed MU.

## Power needed and potential high-power microwave sources to drive a MU

The electron beam in a MU interacts with the backward wave to generate short wavelength radiation^12,18–20^. The backward wave exists in a cavity as a standing wave or a waveguide as a traveling wave. Therefore, the MU can be classified as a cavity-type or waveguide-type MU^21–23^. For the waveguide-type MU, in the ideal case, the entire EM wave energy is confined within the region of the beam aperture $R_b$ in radius. Assuming a planer wave, the relation between the input power *P*, energy *W* inside the MU, the magnetic field *B*, as well as the effective interaction length $L_{eff}$ are given by

3 $$\begin{aligned} \begin{aligned} {W/(\pi R_b^2c/f)}=&{B^2/2\mu _0}={\varepsilon _0E^2/2}, \\ W=&{P/f}, \\ L_{eff}=&{L \times \nu _b/(\nu _b+\nu _{wave})}, \end{aligned} \end{aligned}$$

where *f* is the operating frequency, *L* is the length of the undulator, $\nu _b$ and $\nu _{wave}$ are the velocity of the electron beam and the wave, respectively. The magnetic field as a function of the input power for a waveguide-type undulator, or the flying-type undulator, is

4 $$\begin{aligned} B[\mathrm {T}]=1.63 \sqrt{P [\mathrm {GW}]}/R_b [\mathrm {mm}]. \end{aligned}$$

In a cavity-type MU, at the balanced state where the input power is equal to the Ohmic loss, $W=Q_0P/(2\pi f)$. The magnetic field and the effective interaction length are

5 $$\begin{aligned} \begin{aligned} B[\mathrm {T}]=&0.65 \sqrt{Q_0 P[\mathrm {GW}]} / R_b [\mathrm {mm}], \\ L_{eff}=&L. \end{aligned} \end{aligned}$$

GW-level power is required to achieve a 1.0 T equivalent magnetic field strength for the flying-type undulator with $R_b = 1$ mm, while MW-level input power could reach a similar field strength when a high *Q* cavity is used. The flying-type undulator can have a certain frequency bandwidth (equivalent to continuously tuning the undulator period), while the cavity-type MU can only operate at discrete frequencies due to the high *Q* factor. However the effective interaction length is shorter in the flying-type undulator and its one undesired feature is the magnetic field along the wave propagation direction will taper due to the Ohmic loss in the waveguide.

**Figure 1:**
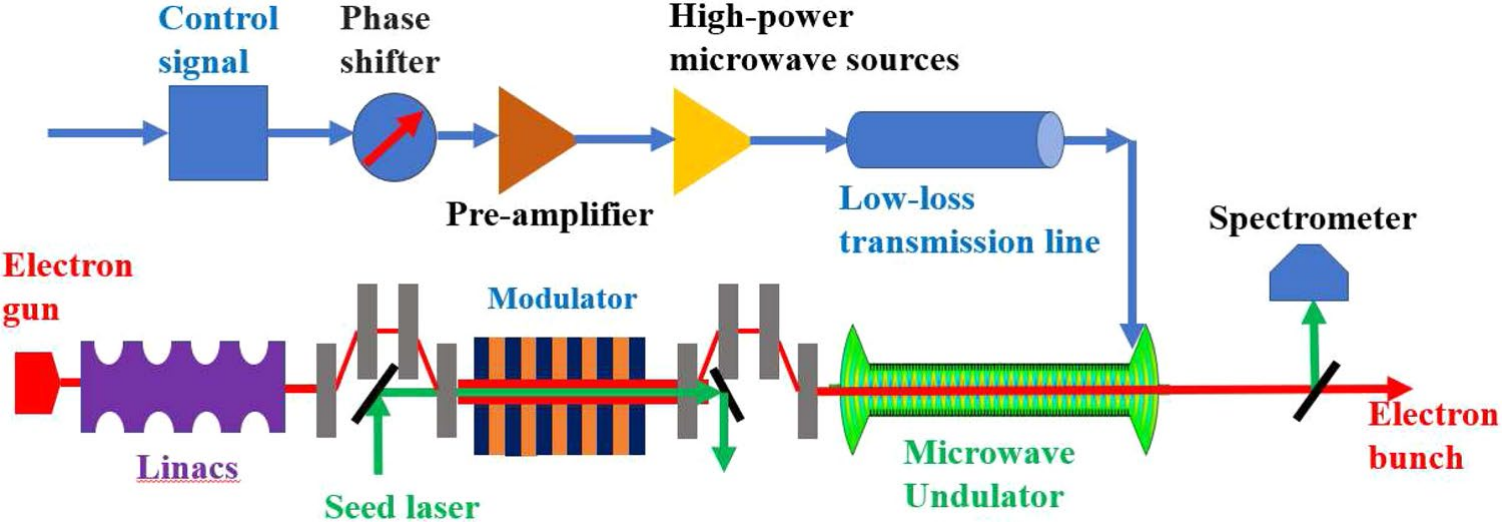
Experimental setup of the microwave undulator.

The cavity-type MU is more attractive in experiments due to the better availability of the MW-level drive sources compared with the GW-level sources. The experimental setup of the MU is shown in Fig. 1. The electron bunch generated from a photocathode RF electron gun will be accelerated by the linac and pre-modulated in a modulator for better interaction with the MU. The MU will be driven by a high-power microwave amplifier through a low-loss transmission line^24^.

**Table 1:** Potential drive sources for MUs.

Frequency	Sources	Specifications
X-band	Klystron	11.424 GHz, 75 MW, 55%^25^
Ka-band	Gyroklystron	35.4 GHz, 15 MW, 33%^26^
	Gyroklystron	30.0 GHz, 15 MW, 40%^27^
	CARM	35.7 GHz, 30 MW, 10%^28^
	Gyrotron	35.0 GHz, 250 MW, 10%^29^
W-band	Gyrotron	94.4 GHz, 5.6 MW, 23%^30^
D-band	Gyrotron	170 GHz, 2 MW, 48%^31^
	CARM	250 GHz, 20 MW, 20%^32^

The choice of the drive source is determined by the techniques of the high-power microwave sources (mainly the vacuum electronic devices) at different operating frequencies, the *Q* factor that the MU structure can achieve, as well as the dynamic of the electron bunch in the MU. Possible high-power microwave sources to drive the cavity-type MUs at different frequencies and the output power efficiencies are listed in Table 1. In X-band or lower frequencies, MW-level RF sources such as klystrons and magnetrons are commercially available and widely used to drive the accelerator structures. The SLAC klystron can achieve an output power of 75 MW at 11.424 GHz^25^ and has been used in MU experiments. To drive shorter period MUs, higher frequency high-power RF sources are therefore more interesting for the future development of the MU. However as the operating frequency increases, the output power from a conventional klystron or magnetron drops dramatically. The gyro-devices, based on the cyclotron resonant maser (CRM) instability and with larger dimensions of the interaction circuit, can achieve high output power at higher frequency^33,34^. A gyroklystron amplifier operating at 36 GHz was designed to generate 3 MW output with a pulse width of 2 ${\mu} \mathrm {s}$ and a repetition rate of 1 kHz^35^. Higher output power from a gyroklystron has been demonstrated in pulsed mode using a coaxial interaction circuit^26^. At the higher frequency range over 100 GHz, the gyrotron oscillator with an overmoded interaction circuit can achieve 1 MW continuous output power at 170 GHz and larger power (5 MW) in the pulsed mode of operation^31^. The Cyclotron Auto-Resonance Maser (CARM), which is also a member of the gyro-device family, can generate high power at higher frequencies however it requires a high quality electron beam at MeV energy^36^. The IAP in Russia has demonstrated 30 MW output power at 35.7 GHz with an efficiency of 10% in short pulse mode^28^. At high frequency, a CARM operating at 250 GHz with 0.5 MW output power was also investigated for the electron cyclotron resonance heating (ECRH) system for the DEMO fusion reactor^32^. Another advantage of using the gyrotrons as the drive source for the cavity-type MU is that different modes can be excited, enabling step tuning of the undulator period. The high power dual frequency gyrotron, for instance, can operate at either 105 GHz or 140 GHz through switching the external magnetic field, while maintaining $\sim$ 1 MW CW output power^37^.

Amplifiers were used in the existing MU experiments. However, in the practical realization of the high-power microwave sources above Ka-band, the oscillators such as gyrotrons are less challenging compared with the amplifiers, such as gyroklystrons or gyrotron travelling wave amplifiers, due to the shorter interaction circuits, no need for the input coupling structures and the higher interaction efficiency. In the following sections, the power requirement is further investigated from a practical design of a MU cavity operating at 36 GHz. And the feasibility of an oscillator as the drive source is studied by investigation of the electron bunch dynamics in the MU.

## Key parameters of the 36 GHz MU cavity

The fundamental differences between a permanent magnet undulator and a microwave undulator have been studied and presented in Refs.^17,23^. In the cavity-type MU, the relativistic electrons see both electric and magnetic fields. If the microwave undulator operates at the desired TE mode with $E_z=0$ and an appropriate electric field polarization with $E_y=0$, the electron’s motion along the *x* axis can be written

6 $$\begin{aligned} \frac{dp_x}{dt} = \frac{e E_0}{2} \left( \frac{\varsigma }{Z_w} +1\right) \cos \left( \omega t + \frac{2\pi z}{\lambda _g}\right) + \frac{e E_0}{2} \left( \frac{\varsigma }{Z_w} -1\right) \cos \left( \omega t - \frac{2\pi z}{\lambda _g}\right) , \end{aligned}$$

where $E_0$ is the peak electric field strength in the MU cavity. $Z_w$ and $\varsigma$ are the wave impedances in the cavity and free space, respectively. Equation (6) contains two terms. The first term is the force from a backward traveling wave, which has an opposite propagating direction to the electron bunch and the second term is the force of a forward traveling wave. From Eq. (6), it can be seen that the side effects of the MU include the undesired modulation of the electrons due to the forward traveling wave and the axial electric field strength if $E_z \ne 0$. To minimize the impact of side effects on the performance of the MU, there are a few criteria for the MU cavity design that need to be taken into account. To achieve a high *Q* factor, the operating mode will have the maximum field strength at the beam path (normally at the cavity center) and minimum field strength on the waveguide wall to reduce the Ohmic loss. The TE-like mode is preferred to maximize the transverse magnetic field and avoid the axial electric field modulating the electron beam. Also to reduce the effect of the forward wave components of the standing wave, the impedance of the forward wave should be close to the vacuum impedance. This means the cavity structure should be overmoded and the operating frequency should be far away from the cut-off frequency of the operating mode in the waveguide of the main body.

**Table 2:** Key parameters of the 36 GHz cavity-type microwave undulator.

Operating mode	$\mathrm{HE}_{11}$
Operating frequency, *f* (GHz)	36.02
*Q* factor, Q	91380
Undulator period, $\lambda _{u}$ (mm)	4.34
Equivalent magnetic field, $B_u$ (T)	1.25
Input power (MW)	50
Overall length (mm)	1048
Undulator parameter, *k*	0.49

**Figure 2:**
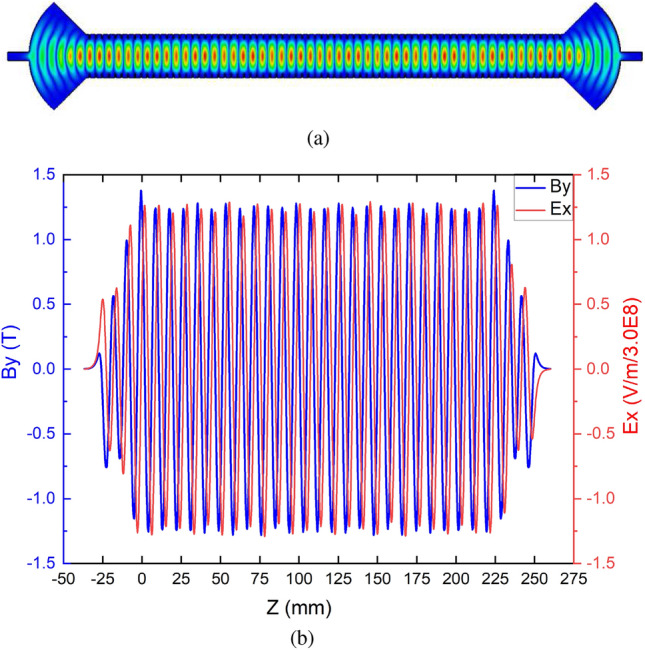
The electric field pattern of the MU cavity with 72 periods of regular corrugation sections (**a**), and the magnetic and electric fields on-axis (**b**).

The quasi-optical $\mathrm{HE}_{11}$ mode, existing in a circularly corrugated waveguide, suits all of these requirements well. With proper geometry parameters, the $\mathrm{HE}_{11}$ mode has a high similarity with the Gaussian mode which has $E_z=0$. Therefore it is also called a quasi-optical mode. In the numerical simulations, the $E_x$ component field strength is ~620 times larger than the $E_z$ field strength. In a FEL, the interaction between the electron bunch and the $E_x$ field is small, around $0.1\%$. The $E_z$ component is very small compared with the electron energy therefore it can be ignored. MUs operating using an $\mathrm{HE}_{11}$ mode have been designed to operate at different frequencies^13,14,17^. An MU operating at 36 GHz was designed for the EU CompactLight XFEL. The corrugated waveguide was designed based on the surface impedance approach, and the coupler structure was designed based on the empirical equations derived from FDTD simulations. Using these two approaches a scalable design was achieved at different operating frequencies and different undulator lengths (different period numbers of the regular corrugation sections). More details can be referred to in^16,17^. Its main properties are shown in Table 2. When the MU structure with an overall length of $\sim$ 1 meter was driven by a 50 MW input power, the equivalent magnetic field was 1.25 T and the undulator period was 4.34 mm. At the same equivalent magnetic field, the drive power needed is nearly proportional to the length of the MU cavity. Simulation results showed the coupler structure had a larger Ohmic loss compared with the regular corrugated waveguide section. The *Q* factor increased as the number of regular corrugation sections of the MU structure increased. A lower drive power will lead to a lower *k* value and the FEL will require a longer MU for interaction to achieve the saturated output radiation. For better visualizing the field inside the MU, Fig. 2a shows the transverse electric field and the electric and magnetic fields along the axis at 72 periods of the regular corrugation sections.

**Figure 3:**
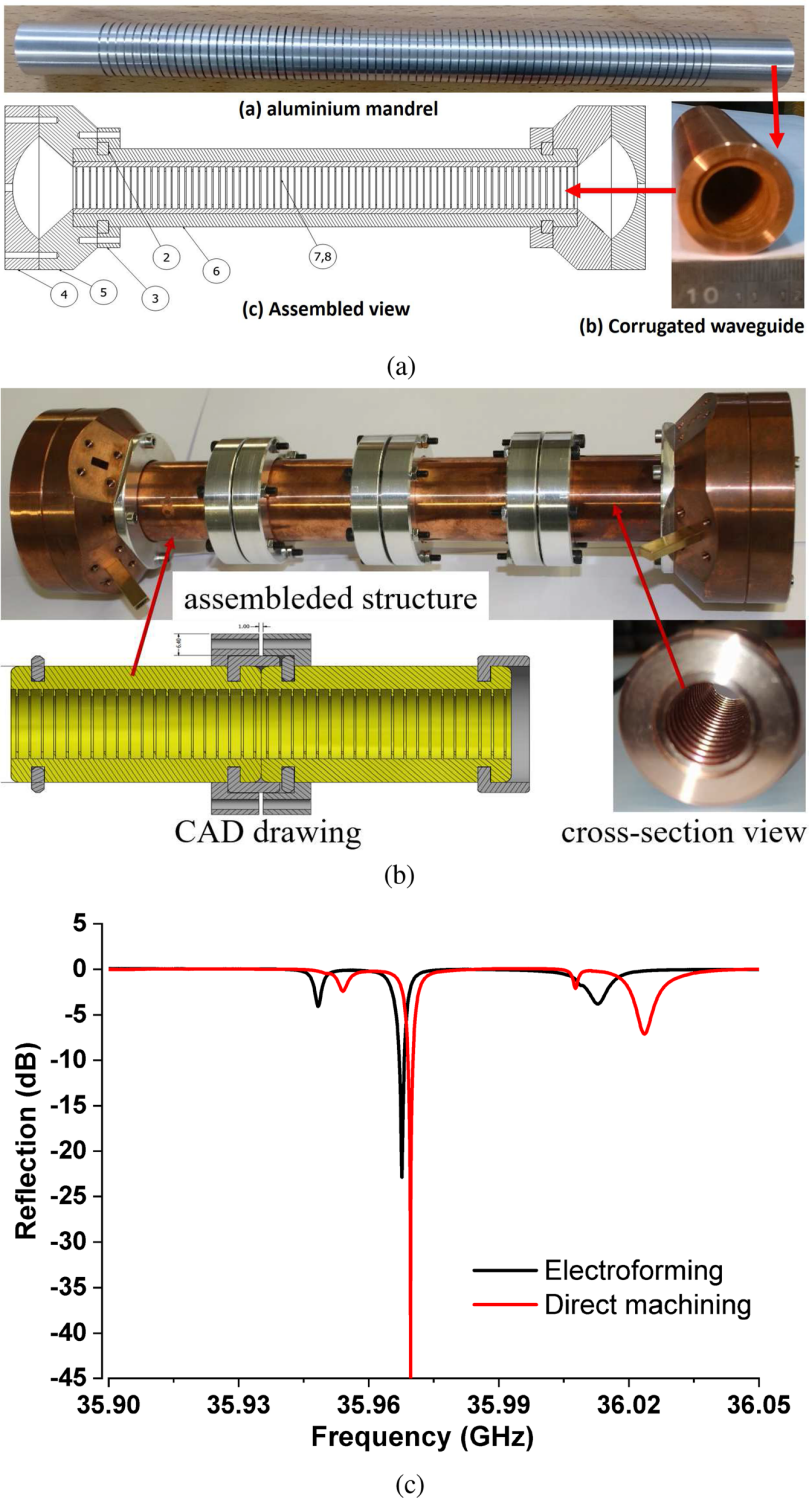
Machined structures of the Ka-band MU by (**a**) electroforming, (**b**) direct machining, and (**c**) the measurement results.

The manufactured MU cavity will have a smaller *Q* factor due to the surface roughness and the machining tolerance. This results in a higher input power to maintain the same equivalent magnetic field. To investigate the differences between the design and manufactured *Q* values, prototypes of the MU cavity with 72 regular periods were manufactured. Both the electroforming method and direct machining were used as shown in Fig. 3. In the electroforming method, the aluminium mandrel, see Fig. 3a, a negative of the corrugated surface, was directly machined from a solid rod using a CNC lathe. Copper was electrodeposited on the mandrel to a minimum radial thickness of 4 mm. The aluminium material was removed in a sodium hydroxide solution resulting in the copper corrugated waveguide. While the corrugated waveguide was directly machined using a CNC lathe. The electroforming method allows the manufacture of a long MU structure in a single piece, which helps to improve the vacuum sealing and reduce the tolerance in the assembly. While the direct machining will require joining a few corrugated waveguide sections into one piece due to the limited length of the machining tool. The other parts of the MU cavity, including the end cap and the coupler, were directly machined using a CNC lathe. All these components were assembled and measured using a Vector Network Analyser (VNA).

The measurement is shown in Fig. 3c, the resonance frequency was 35.967 GHz and the *Q* factor was 53540 for the corrugated waveguide made by the electroforming method. And the values were 35.970 GHz and 71940 for the direct machining method. Both of the resonance frequencies were close to the designed value with an error of less than 0.14%, which indicates the geometrical tolerance is small. The frequency can be slightly increased by machining off a small length of the corrugated waveguide ($\sim$ 0.1 mm) without significantly changing the field pattern and the *Q* factor. The electroforming method has a poorer surface roughness compared with direct machining, and both *Q* factors were smaller than the simulations. Further electropolishing techniques or using diamond tools to directly machine the undulator may help to improve the surface finish and achieve a higher *Q* factor. The $\mathrm{HE}_{11}$ mode has a much higher Q factor compared with the other adjacent modes, which was also confirmed from the eigenmode simulations using CST microwave studio. The frequency separation of the operating mode was about 0.02 GHz. An additional method to improve the *Q* factor is to cool down the MU to a low temperature, or machine the MU cavity from superconducting material^38^. The prototype of the Ka-band MU indicates a high *Q* factor can be achieved close to the designed value, and will not dramatically increase the power needed to drive the MU.

## Electron trajectories in the MU

The electrons in a MU will be modulated by both the transverse electric and magnetic fields. The dynamics of the electrons were therefore studied to avoid the electrons striking the cavity wall since the electric field is large. The maximum drift distance of the electrons will also limit the minimum beam aperture and affect the required drive power of the MU. The motion of a relativistic electron in the electric and magnetic fields is given by

7 $$\begin{aligned} m_0 \frac{d \gamma (v) \vec v}{dt} = q( \vec E(t) + \vec {v} \times \vec B(t) ). \end{aligned}$$

When the interaction efficiency of the FEL is low, the energy loss of the electrons in the undulator is small and was ignored in the calculation. If the space-charge force of the electron bunch is ignored then the trajectories of the electrons can be solved numerically using the electric and magnetic fields exported from the cavity simulations. The trajectories of the electrons inside the MU cavity with a total length $\sim$ 1 m were calculated. The $\vec {E}$ and $\vec {B}$ fields were 3D distributions exported from the eigenmode simulations. Their axial fields were similar to Fig. 2b however with more regular periods.

**Figure 4:**
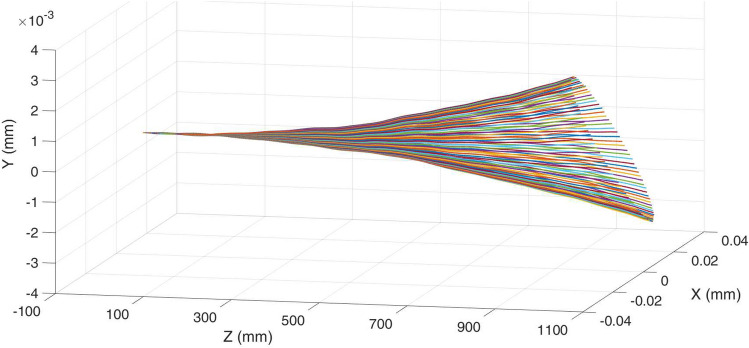
Trajectories of the electrons with 5.5 GeV energy at different phase shifts $\varphi$ inside the MU.

Figure 4 shows the results at a beam energy of 5.5 GeV. The maximum drift distance was nearly proportional to the length of the MU, which limits the maximum length of the MU that can be used. The drift distance on the x-axis is much larger than the y-axis due to the much stronger field strength. Therefore, the motion in the y-axis can be neglected, and only Eq. (8) needs to be solved. Equation (7) can be further simplified with $v=c$ based on the fact that the beam energy is large in high-frequency FELs, and $E_x$, $B_y$ are more than 2 orders of magnitude larger than $E_y$, $E_z$ and $B_x$, $B_z$, respectively, for a linearly polarized wave. Only the motion in the x-axis needs to be solved from the equation

8 $$\begin{aligned} \frac{m_0\gamma _0}{q}\frac{dv_x}{dt}=Re{\left\{ \left( E_x\left( t\right) -cB_y\left( t\right) \right) e^{i\left( \omega t+\varphi \right) }\right\} }, \end{aligned}$$

where $\varphi$ is the phase difference between the microwave field and the injected electrons. $E_x\left( t=0\right)$, and $B_y\left( t=0\right)$ are the electric and magnetic field components in the MU. The results from calculations using Eqs. (7)–(8) were in good agreement. The detailed trajectories showed a small modulation component with a long period, which corresponds to the forward wave component in the cavity. The modulation period matches the long radiation period modulation of $\lambda _{ul}=\lambda _g\lambda _u/(\lambda _g-\lambda _u)$, which is 103.4 mm for the 36 GHz MU.

**Figure 5:**
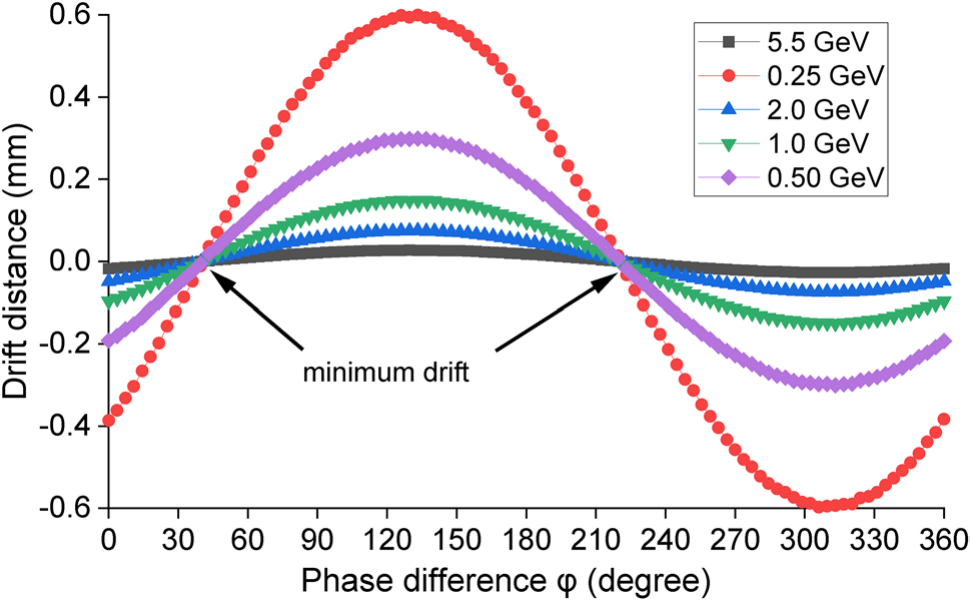
The maximum drift distance as a function of phase shift $\varphi$ at different beam energies.

The maximum drift distance at the exit of the MU is shown in Fig. 5. At high beam energy such as 5.5 GeV, the maximum drift distance is only 0.03 mm. In this case, a high-power oscillator, which is much easier to achieve the required power compared with an amplifier, can be used as the drive source. At lower beam energy, the drift is larger due to the smaller $\gamma$ value. At beam energy of 250 MeV, the maximum drift distance is 0.6 mm, which is still less than the aperture radius 2.0 mm of the 36 GHz MU. A high-power oscillator may still work. However a high-power amplifier that can synchronize the phase between the injected electron beam and the electromagnetic field inside the MU is preferred. The simulations showed that the drift distance can be minimized by choosing proper injection phases $\varphi$. For the designed 36 GHz MU, minimum drift distances can be achieved if $\varphi =41^\circ$ or $221^\circ$. In the cases of beam energy lower than 250 MeV or a longer undulator structure, high-power amplifiers have to be used as the drive sources.

## Electron trajectories and FEL radiation simulated by SPECTRA

The electron trajectories in the MU taking into account the FEL interaction were simulated using SPECTRA^39,40^ with an equivalent static spatial magnetic field profile of the HE$_{11}$ mode in the MU. This also allows simulating the photon flux of the FEL radiation at the same time. Using $t=z/(\beta c)$ in Eq. (8) gives

9 $$\begin{aligned} \frac{\beta m_0 \gamma _0}{q} \frac{dv_x}{dz}=Re{\left\{ \left( E_x\left( z\right) /c-B_y\left( z\right) \right) e^{i\left( \omega t/\beta c+\varphi \right) }\right\} }. \end{aligned}$$

The equivalent spatial magnetic field profile along the *z* axis becomes

10 $$\begin{aligned} B_u\left( z\right) =Re{\left\{ \left( E_x\left( z\right) /c-B_y\left( z\right) \right) e^{i\left( \omega t/\beta c+\varphi \right) }\right\} }. \end{aligned}$$

**Table 3:** Electron bunch parameters.

Maxmimum beam energy (GeV)	5.5
Peak current (A)	5000
Normalized emittance (mm mrad)	0.65
RMS energy spread	0.50%
Repetition rate (Hz)	100

**Figure 6:**
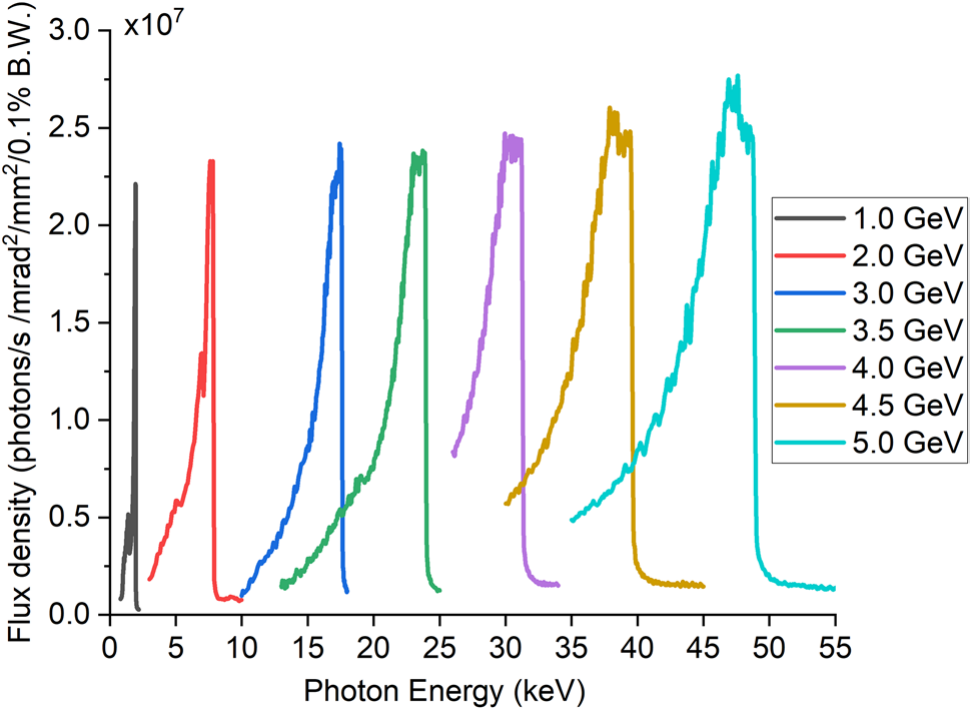
Radiation flux density as the function of radiation energy at different electron beam energies.

**Figure 7:**
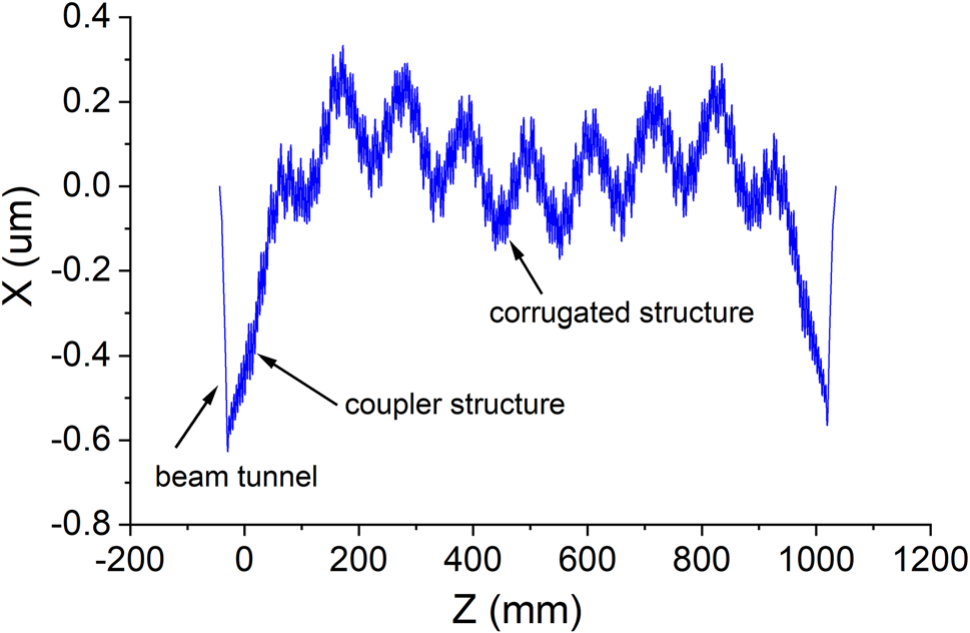
The trajectory of the electron calculated by SPECTRA.

The equivalent on-axis transverse magnetic field at the optimal injection phase was imported into SPECTRA as the user-defined light source. The electron bunch parameters listed in Table 3 were used in the calculations^15^. The flux densities and the photon energies are shown in Fig. 6, as well as the results at lower electron energies. The MU was able to generate a similar level of photon energy at lower beam energy. For example, photon energy of 17.4 keV can be generated with a 3.0 GeV electron energy, compared with a 5.5 GeV electron energy used for a state-of-the-art PMU. Similar photon fluxes will be generated by the MU and PMU if they have the same period length and number along with peak transverse magnetic fields. It is challenging for the MU to achieve a higher photon flux while it is relatively easy for the PMU through increasing the undulator period number since its magnetic field is determined by the magnet structure itself. However for the MU, the Ohmic loss increases proportionally to the number of periods. At the fixed driving power, the longer the undulator, the smaller the peak transverse magnetic field will be. The drive power will be the major factor to limit the photon flux generated from MU. One possible solution can be operating multiple MUs driven by separate microwave sources. This is feasible for the amplifiers using drive sources such as klystrons and gyroklystrons. However it will be a challenge to phase synchronise the microwave radiation in each MU using oscillators such as the gyrotron.

Figure 7 is the result at 3.0 GeV beam energy at the optimal injection phase, which clearly shows the effect in each part of the MU structure. The wave is cut off in the beam tunnel therefore the drift distance linearly increases due to the beam emittance. The electrons were then modulated by the field at the coupler section and had periodic oscillation at the regular corrugation section. The long period seen in Fig. 7 corresponds to the long radiation period which is the same as the previous analysis. The maximum drift distance was about 0.6 ${\mu} \mathrm {m}$ which also agreed with the value solved from Eq. (8).

## Discussion and conclusion

Microwave undulator (MU) has the advantage of short undulator periods by operating at high frequency, which is attractive for compact X-ray FEL facilities. MW-level microwave sources as the driver and high *Q* factor MU cavities are required for realization of a practical cavity-type MU. In this paper, the potential, and a few factors that affect the choice of, high-power microwave sources to drive the cavity-type MUs were discussed. The high *Q* cavity was achieved by the low-loss HE$_{11}$ mode existing in the corrugated waveguide, and prototypes of a short section of the Ka-band MU were machined and tested. The eigen frequency from the measurement is close to the simulation. The *Q* factor is lower due to the imperfect surface roughness. However it is feasible to achieve the design value and ensure no higher drive power is needed by cooling down the cavity structure or machining the cavity from superconducting material. Simulation results of the electron trajectories in the MU showed that at low beam energy, the maximum drift distance of the electrons in the transverse direction is larger. By controlling the injection phase, a close to zero drift distance can be achieved, which requires a high-power amplifier as the drive source. At high beam energy, the drift distance is small therefore an oscillator, which is less challenging compared with an amplifier at the same power level, can be used as the drive source.

For the designed 36 GHz MU, the maximum drift distance was 0.6 mm when the beam energy was 250 MeV. It was smaller than the undulator aperture radius of 2 mm therefore an oscillator, such as a gyrotron oscillator, can be used as the drive source to demonstrate the soft X-ray radiation in a 250 MeV linac. It also opens the feasibility to operate the MU at different modes and step tuning of the radiated photon energy at similar power levels when driven by a high-power multiple-frequency gyrotron.

It should be noted that currently no MW-level Ka-band microwave sources can be used directly to fully demonstrate the performance of the 36 GHz microwave undulator. Different from the well-developed permanent magnetic undulator, the microwave undulator is still in the research stage and the major barrier is the lack of available microwave drive sources. One reason for the slow progress in this area has been the lack of urgent demand through its application. No commercial sources with sufficient stability and long life time has been produced. In recent years, it is encouraging to see increased demand for high-power microwave/millimeter-wave sources, in different applications such as to drive harmonic linearizer for X-ray FELs, or High Q cavities in the search for dark photons, and for heating and current drive in fusion plasma. We hope these demands will bring long-term support to the vacuum electronics community, and allow stable and reliable multi-MW Ka-band gyroklystrons and gyrotrons to be built and tested for MU applications.

## Data Availability

The data that support the findings of this study are available from the corresponding author upon reasonable request.
